# Blood Born miRNAs Signatures that Can Serve as Disease Specific Biomarkers Are Not Significantly Affected by Overall Fitness and Exercise

**DOI:** 10.1371/journal.pone.0102183

**Published:** 2014-07-10

**Authors:** Christina Backes, Petra Leidinger, Andreas Keller, Martin Hart, Tim Meyer, Eckart Meese, Anne Hecksteden

**Affiliations:** 1 Department of Human Genetics, Medical School, Saarland University, Homburg/Saar, Germany; 2 Department of Clinical Bioinformatics, Saarland University, Saarbruecken, Germany; 3 Institute of Sports and Preventive Medicine, Saarland University, Saarbruecken, Germany; Niels Bohr Institute, Denmark

## Abstract

Blood born micro(mi)RNA expression pattern have been reported for various human diseases with signatures specific for diseases. To evaluate these biomarkers, it is mandatory to know possible changes of miRNA signatures in healthy individuals under different physiological conditions. We analyzed the miRNA expression in peripheral blood of elite endurance athletes and moderatly active controls. Blood drawing was done before and after exhaustive exercise in each group. After Benjamini-Hochberg adjustment we did not find any miRNA with significant p-values when comparing miRNA expression between the different groups. We found, however, 24 different miRNAs with an expression fold change of minimum 1.5 in at least one of the comparisons (athletes before vs after exercise, athletes before exercise vs controls and athletes after exercise vs controls). The observed changes are not significant in contrast to the expression changes of the blood born miRNA expression reported for many human diseases. These data support the idea of disease associated miRNA patterns useful as biomarkers that are not readily altered by physiological conditions.

## Introduction

A yet increasing number of microRNAs (miRNAs) is identified in various species including *Caenorhabditis elegans* for which the first report on miRNAs has been published in 1993 [1]. As of now, a total of 24,521 entries representing hairpin precursor miRNAs, expressing 30,424 mature miRNA products in 206 species are deposited in miRBase [2], [3] (release 20), including more than 2,500 different mature miRNAs for *Homo sapiens*. MiRNAs exert their effect by each regulating hundreds of genes post-transcriptionally and are, thus, involved in a manifold of biological processes including proliferation and differentiation. Beyond their physiological role they are also central to many pathological processes [4]. To better understand the physiological and pathological role of miRNAs, many studies do no longer focus on the effects of single miRNAs but analyze the effects of multiple miRNAs in cellular networks. To this end, expression patterns are determined and signatures are derived for various tissues and for specific diseases. While the overwhelming majority of miRNA expression profiles are reported for solid tissues, there are also numerous studies on miRNA profiles in body fluids [5], [6]. In blood, miRNA signatures have been determined both in blood cells and in plasma where they are protected by associated proteins are by inclusion in lipid or lipoprotein complexes [7], [8]. Due to their high stability miRNAs are increasingly explored as future biomarkers for a large variety of human diseases including cancer [9]–[12] but also non cancer diseases [13]. We and others have recently employed standardized operating procedures for measuring blood born miRNA profiles in patients with common adult tumors like lung cancer, childhood cancer like Wilms tumor, and multiple non cancer diseases like multiple sclerosis, chronic obstructive pulmonary disease (COPD), and acute myocardial infarction [14]–[16]. In a comparative study of 863 microRNAs in 454 blood samples from human individuals with 14 different diseases, we found on average for each disease 103 significantly deregulated miRNAs (*P*<0.05; *t*-test after Benjamini-Hochberg adjustment) [17]. In addition, we recently identified miRNA expression profiles in blood samples of healthy individuals including long-lived individuals with a mean age of 96.4 years. The miRNA expression data revealed a distinct separation between the long-lived individuals and younger controls (P-value <10^−5^) [18]. To better understand the meaning of altered miRNA expression pattern both in diseased and in healthy individuals, it is important to also analyze the effect of physiological challenges to the healthy individuals. As of now there are only very few studies analyzing miRNA expression changes in healthy individuals under various conditions [19], [20]. Recently, studies have analyzed the changes of miRNA expression in peripheral blood mononuclear cells [21] and neutrophils after acute exercise [22], [23]. Here we set out to determine the impact of acute exercise and long-term exercise training on miRNA expression in peripheral blood of elite endurance athletes and matched controls before and after exhaustive exercise.

## Material and Methods

### Participants

12 elite endurance athletes (6 males, 6 females; 10 triathletes, 2 cyclists) and 12 age- and sex-matched, moderately active controls participated in the present study. Subject characteristics are summarized in Table 1. All athletes compete on an international level and were mostly recruited at the Olympic training center Saarbrücken. Two of the study subjects participated in the 2012 Olympic Games. Controls were matched for sex and age (±2 years) and are engaged in recreational physical activties only.

**Table 1:** Anthropometric data and physical capacity.

	Athletes	Controls	p-value
Sex (♀/♂)	6/6	6/6	n.a.
Age (years)	24±5	25±3	0.73
Heigth (cm)	176±10	173±9	0.52
Body mass (kg)	65±10	69±12	0.41
BMI (kg/m^2^)	21±1	23±3	**0.04**
Pmax (W/kg)	5,2±0,5	3,5±0,7	**<0.01**

BMI  =  body mass index; Pmax  =  maximal workload attained during the stepwise incremental exercise test.

means ± standard deviation; Statistical testing of differences between groups: 1-way ANOVA.

All subjects gave written informed consent prior to participation. The study was approved by the local ethics commitees (Ärztekammer des Saarlandes; ID 115/12).

### Blood sampling

Participants reported to the laboratory between 8 and 10 a.m. after abstaining from physical exercise for at least 36 hours (A fasting period was not required to enable the recruitment of elite athletes). After a supine rest period of 10 minutes venous blood samples were collected from the antecubital vein by standard techniques. Samples for the determination of miRNA expression were collected in special tubes (PAXgene blood RNA tube, Becton Dickinson, Germany) and stored at −20°C until analysis. Post-exercise blood samples were collected in the same way 30 min after cessation of exercise.

### Exercise testing protocol

An exhaustive, stepwise exercise test was conducted on a calibrated cycle ergometer (Excalibur Sport, Lode B.V., Groningen, Netherlands). Initial load was 50 W for women and 100 W for men. Step duration and increment were 3 min and 50 W, respectively. Verbal encouragement was given to all subjects during the final stages of the exercise test. Capillary blood samples for the determination of blood lactate concentration were taken from the hyperemizied earlobe at rest, during the last 15 seconds of each step as well as 1, 3, 5, 7 and 10 min after cessation of exercise. Samples were immediately hemolyzed and analysis carried out using an enzymatic-amperometric system (Super GL, Greiner, Flacht, Germany).

Objective criteria of exhaustion (maximal blood lactate concentration of >8 mmol/l and maximal heart rate of >200-age (years)) were met by all subjects.

### RNA Isolation

Total RNA including miRNA was isolated using the PAXgene Blood miRNA Kit (Qiagen GmbH, Hilden, Germany) following the manufacturer's recommendations. Isolated RNA was stored at −80°C. RNA integrity was analysed using Bioanalyzer 2100 (Agilent Technologies, Böblingen, Germany) and concentration and purity was measured using NanoDrop 2000 (Thermo Fisher Scientific, Schwerte, Germany).

### miRNA Microarray

Microarray analysis was peformed with samples from 8 endurance atheletes and 8 controls according to the manufacturer's instructions using SurePrint 8×60K Human v16 miRNA microarrays (Agilent, CatNo G4870A) that contain 40 replicates of each of the 1,205 miRNAs of miRBase v16 (http://www.mirbase.org/). In brief, a total of 100 ng total RNA was processed using the miRNA Complete Labeling and Hyb Kit to generate fluorescently labeled miRNA. This method involves the ligation of one Cyanine 3-pCp molecule to the 3′ end of a RNA molecule with greater than 90% efficiency. First, the RNA is dephosphorylated using Calf Intestinal Alkaline Phosphatase (CIP). After the dephosphorylation step, dimethylsulfoxide (DMSO), which is an effective RNA denaturant, is added to the samples and the RNA is heat denaturated to minimize the effect of structure and sequence differences among miRNAs. Using T4 RNA ligase and a 3′, 5′-cytidine bisphosphate which is labelled by a cyanine dye at its 3′ phosphate (pCp-Cy3) miRNA molecules with an additional 3′-cytidine and exactly one cyanine dye on its 3′end are produced. After the labelling reaction, the mixture is dryed in a vacuum centrifuge and resuspended in the hybridization mixture containing hybridization buffer and blocking reagent. Then the microarrays were loaded and incubated for 20 h at 55°C and 20 rpm. To check if the labelling and hybridization was successful, labeling and hybridization spike-in controls were added in the appropriate steps. After several washing steps microarrays were scanned with the Agilent Microarray Scanner at 3 microns in double path mode. Microarray scan data were further processed using Feature Extraction software.

### Statistical data evaluation of microarrays

Using the raw totalProbeSignals generated by the Agilent Feature Exraction Software, we applied quantile normalization and log2 transformed the expression values. After that we kept only miRNAs that were expressed (flagged as detected from the Feature Extraction Software) in all samples of at least one group in a comparison. We carried out parametric t-test (two-tailed, paired for samples from same individuals before and after exercise, unpaired otherwise) for each miRNA separately to detect miRNAs that show different behavior in different groups of blood donors. The resulting p-values were adjusted for multiple testing by Benjamini-Hochberg adjustment [24], [25].

### Quantitative Real Time Polymerase Chain Reaction (qRT-PCR)

The qRT-PCR was performed with samples from 12 endurance atheletes and 12 controls. Using the miScript PCR System (Qiagen), we analyzed the expression of three miRNAs that were expressed in all 32 samples from the microarray experiment, namely hsa-miR-181a-5p, hsa-let-7c, and hsa-miR-24-3p. RNU48 was used as endogenous control [26]–[30]. In brief, 100 ng RNA was reverse transcribed using miScript HiSpec Buffer following manufacturers recommendations, but in a final vomlume of 10 µl. The PCR was run in a final volume of 10 µl with 2 µl diluted (1∶100) cDNA.

## Results

### Subject characteristics

Anthropometrical and physical performance data are summarized in Table 1. A significantly lower BMI in elite athletes is caused by slight numerically differences in body weight and height in opposite directions. Maximum performance values for the two groups substantiate the expected contrast in physical capacity.

### MicroRNA expression analysis

We analysed the expression of 1,205 different miRNAs in blood of 8 elite endurance athletes and 8 age and sex matched controls. Out of 1,205 tested miRNAs 901 miRNAs were not expressed in any of the analyzed blood samples. A total of 154 miRNAs were expressed in all 32 analyzed blood samples. In detail, in the blood of controls obtained before and after exercise we detected 167 and 161 miRNAs, respectively, and in the blood of athletes obtained before and after exercise we detected 172 and 173 miRNAs, respectively. Overall, we found 173 miRNAs that were expressed in more than 90% of all analyzed samples. Table 2 lists the number of miRNAs that were not expressed in any sample and expressed in only one sample, two samples, etc. of each group.

**Table 2:** Number of miRNAs expressed in 0–8 samples of each group.

	0	1	2	3	4	5	6	7	8	in minimum 1 sample
controls before	940	20	15	13	12	17	7	14	167	265
controls after	917	33	15	17	13	9	20	20	161	288
athletes before	935	20	12	16	8	9	14	19	172	270
athletes after	929	25	14	9	13	13	14	15	173	276

We grouped all blood samples according to whether they were obtained from controls or endurance athletes and whether they were drawn before or after the exertion. Furthermore, we grouped the samples according to the time point of blood withdrawal (before or after exercise) regardless of the study participants. We did not find any miRNA yielding significant p-values after Benjamini-Hochberg adjustment for the comparisons athletes before vs after exercise, athletes before vs controls before exercise, and athletes after vs controls after exercise. The only significant miRNA hsa-miR-320b after adjustment was found for the comparisons controls before vs controls after exercise (fold change: 1.4) and all samples before vs all samples after exercise (fold change 1.6).

**Figure 1:**
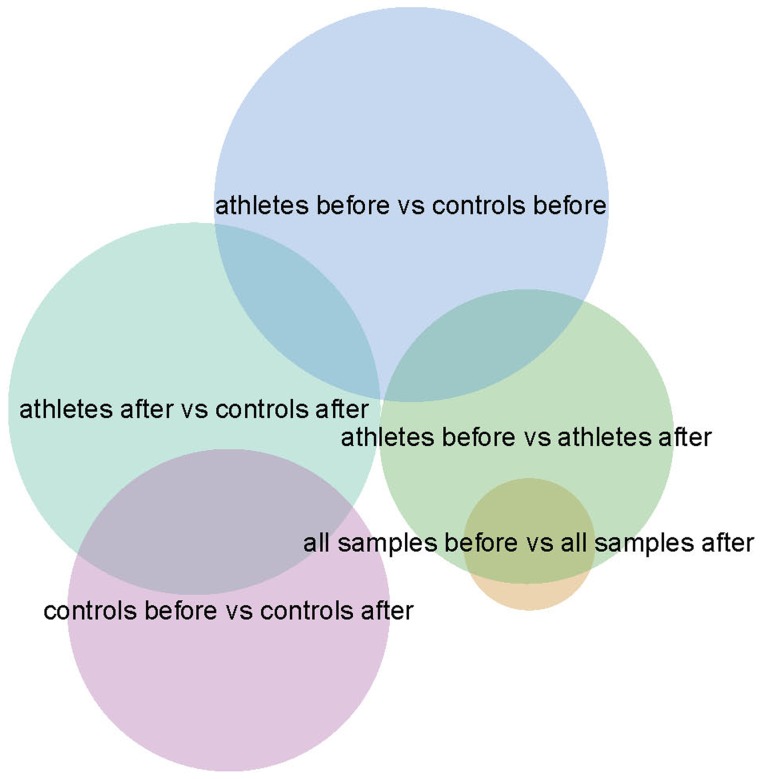
Venn diagram of the miRNAs with fold changes >1.5 in the different group comparisons.

For further analyses, we extracted all miRNAs that showed an absolute expression fold change of at least 1.5 in any of the above mentioned comparisons. This revealed a total of 29 miRNAs (including 24 different miRNAs). Table 3 lists these miRNAs with fold changes >1.5 for the different comparisons. Figure 1 shows a venn diagram indicating the overlapping miRNAs between the different comparisons.

**Table 3:** miRNAs with fold change >1.5 in the different comparisons.

		median group 1	median group 2	logqmedian	median fold change	ttest rawp	AUC	
athletes	hsa-miR-494	5,184173398	5,896861896	−0,712688498	−1,638855321	0,097997900	0,7578125	up
before vs	hsa-miR-150	10,72359337	11,4038118	−0,680218429	−1,602382343	0,268030702	0,6328125	up
althletes after	hsa-miR-3656	7,282186154	6,694879568	0,587306586	1,502439175	0,057284503	0,3828125	down
	hsa-miR-144*	6,180073597	5,502584859	0,677488738	1,599353377	0,560463180	0,3984375	down
	hsa-miR-320b	10,00967385	9,325617772	0,684056075	1,60665044	**0,030736681**	0,359375	down
athletes	*hsa-miR-563*	2,311943693	3,209581359	−0,897637666	−1,863012902	0,171494856	0,75	up
before vs	*hsa-miR-3180-3p*	3,69575586	4,352516391	−0,656760531	−1,576538641	0,30978821	0,609375	up
controls	hsa-miR-629	3,389998045	2,795845641	0,594152404	1,509585431	**0,043596785**	0,2109375	down
before	hsa-let-7b	11,80686722	11,15338943	0,653477789	1,572955427	0,166432114	0,296875	down
	hsa-miR-182	3,920389491	3,258942332	0,661447159	1,581668391	0,194567263	0,3203125	down
	hsa-let-7c	7,881850981	7,204856236	0,676994746	1,598805838	**0,027465882**	0,1640625	down
	*hsa-miR-98*	4,53605938	3,81236171	0,72369767	1,651409226	0,050531661	0,203125	down
	hsa-miR-144*	6,180073597	5,406467128	0,773606469	1,709537973	0,675212368	0,4921875	down
	hsa-miR-183	6,461844278	5,678446337	0,783397941	1,721179952	0,0590943	0,234375	down
athletes after	*hsa-miR-27a*	3,65966376	4,551620012	−0,891956253	−1,855690681	0,346005439	0,6484375	up
vs controls	*hsa-miR-1274a*	3,539826718	4,399130323	−0,859303605	−1,814162396	0,233977017	0,6484375	up
after	hsa-miR-4286	4,34102114	5,161308005	−0,820286864	−1,76575706	0,281432877	0,65625	up
	*hsa-miR-148a*	3,115085426	3,930077176	−0,814991751	−1,759288093	0,203644838	0,71875	up
	hsa-miR-30e	4,214221792	4,910975925	−0,696754133	−1,620853986	0,80986352	0,5	up
	*hsa-miR-98*	4,308581136	3,706456759	0,602124377	1,517950112	0,220850161	0,296875	down
	hsa-miR-181a	6,218547498	5,588206679	0,630340819	1,54793063	**0,039032053**	0,1953125	down
	hsa-miR-197	6,814805493	6,130665142	0,684140351	1,606744296	0,354239372	0,359375	down
	hsa-miR-320b	10,00967385	9,325617772	0,684056075	1,60665044	**0,00004845**	0,3515625	down
all samples before vs all samples after	hsa-miR-1260	3,866717805	4,579511597	−0,712793792	−1,638974936	0,185206743	0,75	up
controls	hsa-miR-30e	4,222505424	4,910975925	−0,688470501	−1,611574073	0,210651178	0,6015625	up
before vs	*hsa-miR-27a*	3,906470607	4,551620012	−0,645149405	−1,563901235	0,138004582	0,65625	up
controls after	hsa-miR-320d	9,473326877	8,858763331	0,614563545	1,531094729	**0,001930516**	0,28125	down
	hsa-miR-320e	8,814483484	8,194836789	0,619646696	1,536498859	**0,015668754**	0,25	down
	hsa-miR-320c	8,609153145	7,937844648	0,671308497	1,592516696	**0,002253772**	0,2109375	down

miRNAs in italics are not expressed in all 32 tested samples. Numbers in bold are statistically significant p-values.

We found five miRNAs that were differentially expressed based on the fold change criterion in blood samples of the endurance athletes due to heavy exercise. Of those, 2 miRNAs were upregulated and three miRNAs were downregulated. In the control group, heavy exercise resulted in >1.5 fold deregulation of six miRNAs, with three upregulated and three downregulated miRNAs. Interestingly, we found no overlap between the deregulated miRNAs of those two comparisons. Most deregulated miRNAs were found for the comparison of endurance athletes and controls before exercise and likewise for the comparison of endurance athletes and controls after exercise. This fold change based analysis shows that more miRNAs are differentially expressed between controls and endurance-trained athletes than between probands before and after the exhaustive exercise.

We obtained nine miRNAs differentially expressed between endurance athletes and controls comparing all samples obtained before exercise, and also nine miRNAs differentially expressed between endurance athletes and controls comparing all samples obtained after exercise. The comparison of all samples obtained before versus all samples obtained after exercise revealed only one deregulated miRNA.

**Figure 2:**
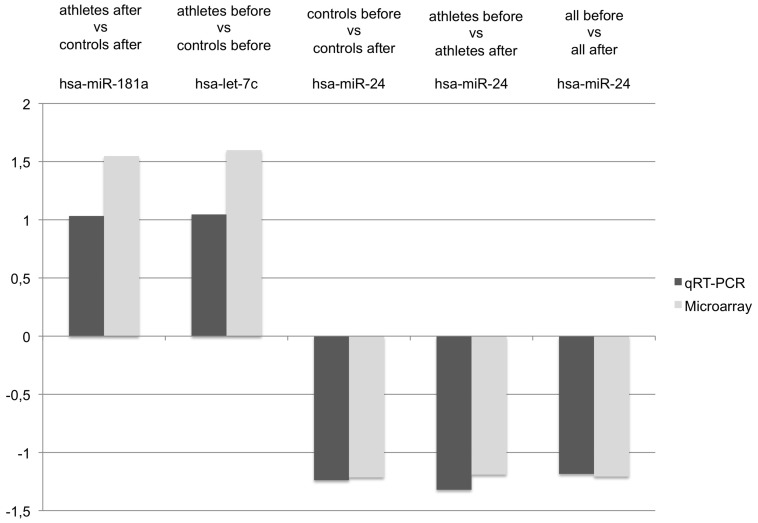
Comparisons of the fold change of the qRT-PCR and the microarray results. Comparison of the fold changes of three miRNAs found deregulated in different comparisons in the microarray experiment and the corresponding qRT-PCR results. A fold change means an upregulation of the respective miRNA in the first group of the comparison; a negative fold change means a downregulation of the respective miRNA in the first group of the comparison.

To validate the microarray results we performed qRT-PCR using blood samples obtained before and after exercise from 12 endurance athletes and 12 controls. Of those, samples from 8 endurance athletes and 8 controls were already used for microarray analysis. In detail, we analyzed the expression of hsa-miR-181a that was >1.5 fold more expressed in controls compared to endurance athletes after exercise (p-value 0.0390), and of hsa-let-7c that was >1.5 fold more expressed in controls compared to endurance athletes before exercise (p-value 0.0275). Furthermore, we analyzed hsa-miR-24, that was only about 1.2 times more expressed in endurance athletes before vs endurance athletes after exercise (p-value 0.0257), controls before vs controls after exercise (p-value 0.0319), and all samples before vs all samples after exercise (p-value 0.0011). These three miRNAs were expressed in all 32 analyzed blood samples according to the microarray analysis. The results of the qRT-PCR were also not significant, but confirmed the results of the microarrays. Although the fold changes were smaller than those revealed by microarray, the direction of regulation was the same (see Figure 2).

### Target gene analysis

We extracted the predicted targets from miRDB v4.0 [31] for the 25 miRNAs with fold changes >1.5 for the different comparisons and performed an over-representation analysis using GeneTrail [32]. We found 5 KEGG categories with p-values below 0.05 and FDR adjustment.

We compared the list of predicted targets for the 25 miRNAs with gene expression data of white blood cells published by Büttner et al [33]. In their study they identified 39 upregulated transcripts of 30 different genes and 7 downregulated transcripts of 4 different genes as exercise marker genes. We selected these transcripts and examined whether they are possible targets of the miRNAs that were found to be deregulated in different comparisons in our study. This analysis revealed 35 transcripts from 13 different genes, namely the upregulated *CGI-58, TGFA, F5, SLC2A3, REPS2, MME, MOSC1, ABAT, TRIB1*, and *IL1RAP* and the downregulated *PTGDR, FAM115A*, and *YES1*, that might be targeted by the deregulated miRNAs identified in our study (see Table 4).

**Table 4:** Genes deregulated after exercise extracted from Büttner et al. [33] and the miRNAs deregulated in different comparisons in our study that are predicted to regulate these genes.

miRNA	Comparison	Transcript	GeneID	GeneName	gene Regulation	miRNA Regulation
hsa-miR-183-5p	athletes before vs controls before	NM_000663	18	ABAT	up	down
hsa-miR-183-5p	athletes before vs controls before	NM_020686	18	ABAT	up	down
hsa-miR-183-5p	athletes before vs controls before	NM_001127448	18	ABAT	up	down
hsa-miR-30e-5p	athletes after vs controls after/controls before vs controls after	NM_016006	51099	CGI-58	up	up/up
hsa-miR-181a-5p	athletes after vs controls after	NM_000130	2153	F5	up	down
hsa-miR-494	athletes before vs athletes after	NM_000130	2153	F5	up	up
hsa-miR-4286	athletes after vs controls after	NM_014719	9747	FAM115A	down	up
hsa-miR-629-5p	athletes before vs controls before	NM_014719	9747	FAM115A	down	down
hsa-miR-181a-5p	athletes after vs controls after	NM_001167930	3556	IL1RAP	up	down
hsa-miR-181a-5p	athletes after vs controls after	NM_134470	3556	IL1RAP	up	down
hsa-miR-197-3p	athletes after vs controls after	NM_001167930	3556	IL1RAP	up	down
hsa-miR-197-3p	athletes after vs controls after	NM_134470	3556	IL1RAP	up	down
hsa-miR-27a-3p	athletes after vs controls after/controls before vs controls after	NM_001167928	3556	IL1RAP	up	up/up
hsa-miR-27a-3p	athletes after vs controls after/controls before vs controls after	NM_001167929	3556	IL1RAP	up	up/up
hsa-miR-27a-3p	athletes after vs controls after/controls before vs controls after	NM_002182	3556	IL1RAP	up	up/up
hsa-miR-494	athletes before vs athletes after	NM_001167928	3556	IL1RAP	up	up
hsa-miR-494	athletes before vs athletes after	NM_001167929	3556	IL1RAP	up	up
hsa-miR-494	athletes before vs athletes after	NM_002182	3556	IL1RAP	up	up
hsa-miR-181a-5p	athletes after vs controls after	NM_007287	4311	MME	up	down
hsa-miR-181a-5p	athletes after vs controls after	NM_000902	4311	MME	up	down
hsa-miR-181a-5p	athletes after vs controls after	NM_007289	4311	MME	up	down
hsa-miR-181a-5p	athletes after vs controls after	NM_007288	4311	MME	up	down
hsa-miR-27a-3p	athletes after vs controls after/controls before vs controls after	NM_022746	64757	MOSC1	up	up/up
hsa-miR-27a-3p	athletes after vs controls after/controls before vs controls after	NM_000953	5729	PTGDR	down	up/up
hsa-miR-30e-5p	athletes after vs controls after/controls before vs controls after	NM_000953	5729	PTGDR	down	up/up
hsa-miR-1260a	controls before vs controls after	NM_000953	5729	PTGDR	down	up
hsa-miR-181a-5p	athletes after vs controls after	NM_004726	9185	REPS2	up	down
hsa-miR-181a-5p	athletes after vs controls after	NM_001080975	9185	REPS2	up	down
hsa-miR-4286	athletes after vs controls after	NM_004726	9185	REPS2	up	up
hsa-miR-4286	athletes after vs controls after	NM_001080975	9185	REPS2	up	up
hsa-miR-182-5p	athletes before vs controls before	NM_006931	6515	SLC2A3	up	down
hsa-miR-148a-3p	athletes after vs controls after	NM_003236	7039	TGFA	up	up
hsa-miR-148a-3p	athletes after vs controls after	NM_001099691	7039	TGFA	up	up
hsa-miR-150-5p	athletes before vs athletes after	NM_025195	10221	TRIB1	up	up
hsa-miR-182-5p	athletes before vs controls before	NM_005433	7525	YES1	down	down

For certain genes several transcripts are listed. The direction of regulation of the miRNAs and the target genes is given in the last two columns.

### Comparison of the miRNA expression profile of athletes with published data

**Figure 3:**
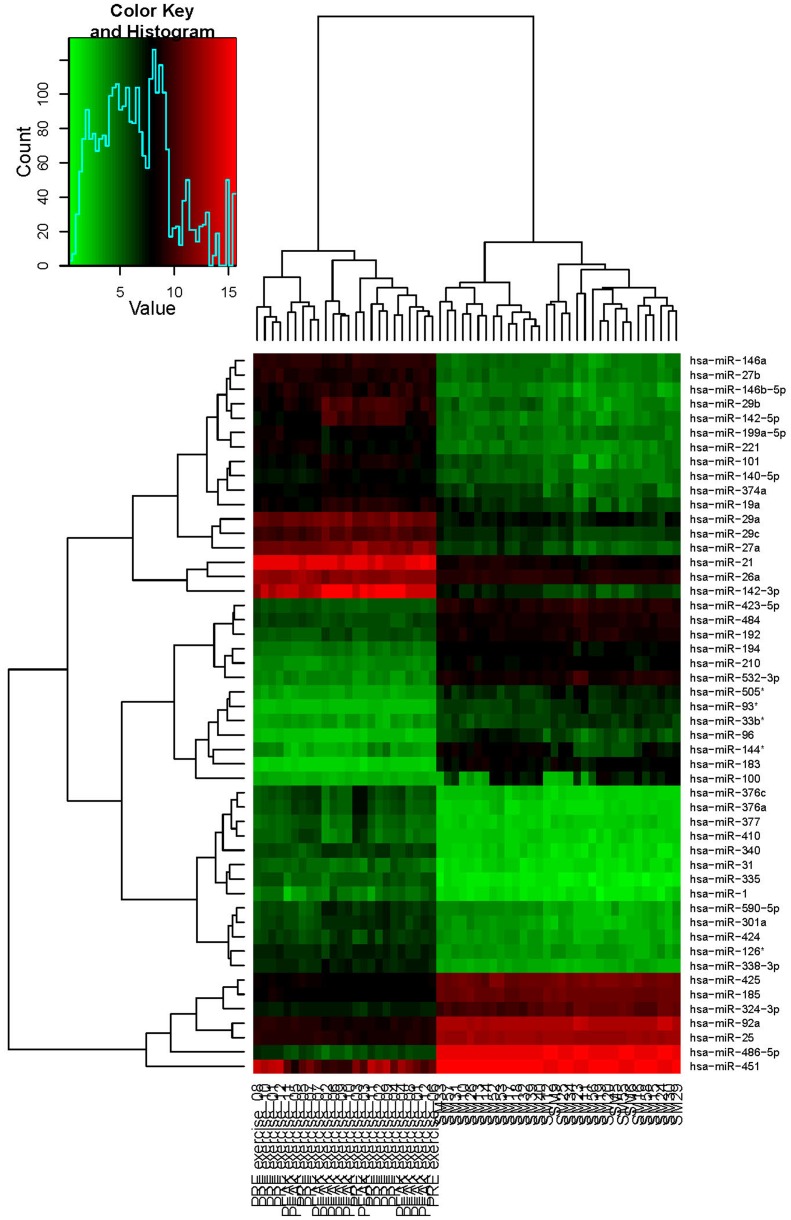
Heatmap for the 50 miRNAs with highest variance and all samples analyzed in the study by Radom-Aizik [21] and in our study.

We extracted the microarray data from Radom-Aizik et al. [21] deposited in the GEO database (GSE28745) and compared their results to ours. A total of 17 out of 34 miRNAs deregulated in the Radom-Aizik study were expressed in all of the 32 tested samples of our study, 10 miRNAs were expressed in 22 to 31 samples, one miRNA was only expressed in two samples, and six miRNAs were not expressed in any of the tested samples. Only miRNA hsa-miR-181a, significantly deregulated in the Radom-Aizik study was also more than 1.5-fold deregulated in our study. We have to point out that the study designs differed in that Radom-Aizik et al. analyzed the expression of 723 miRNAs in peripheral mononuclear cells (PBMC) from untrained individuals before and after exercise whereas we analyzed the expression of 1205 miRNAs in whole blood of athletes before and after exertion. We found an overlap of 700 miRNAs present on both microarray types. To determine only the influence of the different sample types (PBMC vs whole blood) and participants (untrained vs athletes) independent of the exercise protocols, we compared the microarray data of both studies. Of the 700 miRNAs present on the microarrays of both studies, 202 were expressed in all samples from Radom-Aizik before exercise and 125 were expressed in our study in all 8 endurance athletes and 119 were expressed in all 8 control samples before physical activity. Out of the 202 miRNAs expressed in all samples before exercise from Radom-Aizik, a total of 53 and 52 miRNAs were not expressed in our samples from endurance and controls before exercise, respectively. Figure 3 shows a heatmap for the 50 miRNAs with highest variance and all samples obtained before and after exercise analyzed in our study and by Radom-Aizik et al. and clearly illustrates the differences between both studies relying on the different study designs.

## Discussion

In our study we set out to examine the effect of exhaustive exercise on the miRNome of whole blood of both elite endurance athletes and sex and age matched moderately active controls. We identified a total of 25 miRNAs that were >1.5-fold differentially expressed between blood samples obtained from elite endurance athletes and controls. However, their deregulation was in most cases not statistically significant after adjustment. In detail, we found five miRNAs deregulated after exhaustive exercise in elite endurance athletes, namely hsa-miR-144*, hsa-miR-150, hsa-miR-320b, hsa-miR-3656, and hsa-miR-494. Some of those miRNAs have already been reported to be involved in haematopoiesis. The miR-451/miR-144 cluster has been found to play a crucial role in erythropoiesis and the miRNA hsa-miR-150 is involved in myelopoiesis, megakaryopoiesis, B- and T-cell development, and NK cell development [34]–[36]. Six miRNAs were altered in expression due to exhaustive exercise in controls, namely hsa-miR-1260, hsa-miR-27a, hsa-miR-30e, hsa-miR-320c, hsa-miR-320d, and hsa-miR-320e. Besides others, the miR-30 family regulates the expression of *BCL6* and *PRDM1* that are involved in interleukine and interferone production [35]. Interestingly, there was no overlap in the miRNAs deregulated after exercise in endurance athletes and controls. However, samples from elite endurance athletes and controls in rest differed in nine miRNAs. After exhaustive exercise the differences in eight of nine miRNAs disappeared, but eight additional other miRNAs were differentially expressed. The one overlapping miRNA hsa-miR-98 is part of the let-7 cluster and is deregulated in activated human platelets [36].

The identification of miRNAs that are indicative for the individual fitness level or training load and the knowledge on the target genes that are regulated by those miRNAs might give insight into the physiological aspects connected with sports. A target gene analysis for the 25 miRNAs that were >1.5-fold deregulated in the different comparisons of our study revealed influences on five KEGG pathways. Most interestingly, we found a significant enrichment (p-value 0.0018) of genes involved in the neurotrophin signaling pathway. Neurotrophins are involved in the proliferation, differentiation, survival and death of neuronal and non-neuronal cells and are implicated in neurodegenerative disorders [37]. It is known that physical activity positively influences mental function. Several neurotrophic factors are increased due to physical activity [38].

Radom-Aizik et al. analyzed the expression of miRNAs in peripheral blood mononuclear cells (PBMC) obtained from untrained individuals before and after exercise and identified 34 significantly >1.2-fold deregulated miRNAs. We only found a small overlap of deregulated miRNAs identified in their study compared with our results. This might be explained by the differences in study design, as we analyzed the miRNA expression pattern of whole blood obtained from athletes. As PBMC account for only about 1% of cells in whole blood it is conceivable that slight changes in miRNA content of leucocytes might be masked. These discrepancies are also underlined by the heatmap of the 50 miRNAs with highest variance and all samples of our study and the Radom-Aizik study. The heatmap is separated into 4 clusters with one cluster comprising miRNAs that are known to be enriched in red blood cells, namely hsa-miR-451, hsa-miR-486-5p, and hsa-miR-92a [39] or involved in erythropoiesis, namely hsa-miR-185 [40]. On the other hand, our findings might also indicate that heavy exhaustive exercise has a lower effect on athletes than on untrained individuals, suggesting a general influence of the fitness level on the miRNA expression pattern in blood. This hypothesis is underlined by the findings that the samples obtained before exercise from endurance athletes and controls differ in 9 miRNAs and that we did not find any overlap between the comparisons of samples obtained before and after exercise from endurance athletes and controls, respectively. We hypothesize that the individual fitness level impacts the dimension of miRNA expression changes due to exercise.

The observed changes of the miRNA expression in the present study were, however, not statistically significant. In a multicenter study we previously compared the blood expression profiles of 863 miRNAs in 454 analyzed blood samples from 14 different human diseases and found disease-specific alterations of the miRNA pattern [17]. In detail we found miRNA signatures for lung cancer, prostate cancer, pancreatic ductal adenocarcinoma, melanoma, ovarian cancer, gastric tumors, Wilms tumor, pancreatic tumors, multiple sclerosis, chronic obstructive pulmonary disease (COPD), sarcoidosis, periodontitis, pancreatitis, and acute myocardial infarction. For each disease we found an average of 103 deregulated miRNAs (*P*<0.05; *t*-test after Benjamini-Hochberg adjustment). The according blood-borne ‘miRNome’ data are deposited in the Gene Expression Omnibus and available at http://genetrail.bioinf.uni-sb.de/wholemirnomeproject/. In contrast to the diseases, the miRNome of healthy individuals does not seem to be significantly changed neither by acute exhaustive exercise nor by the long-term effects of training performed by elite endurance athletes.

## Conclusions

Although the overall fitness and the exercise do seem to impact the miRNA expression level in human blood cells, the observed changes are not comparable to the expression changes found for human diseases. The deregulation of the miRNAs identified in the present study was in most cases not statistically significant after adjustment. MiRNA patterns appear to be rather robust at least against the influence of training effects. This observation lends further support to the employment of miRNA signatures as disease associated biomarkers.
